# Incidence and Molecular Detection of Genotypes for Giardia lamblia Isolated From Children in Zakho District, Duhok Province, Kurdistan Region, Iraq

**DOI:** 10.7759/cureus.66626

**Published:** 2024-08-11

**Authors:** Ahmed Basheer Mohammed

**Affiliations:** 1 Molecular Biology/Biology Department, Faculty of Science University of Zakho, Zakho, IRQ

**Keywords:** zakho, children, infection rate, pcr-rflp, giardia

## Abstract

*Giardia lamblia* is a significant intestinal protozoan in humans worldwide, with a high incidence of infection in developing countries, particularly among children. Molecular analysis has identified eight assemblages (A to H), with A and B more frequently associated with human infections. Regardless of its importance, to the best of our knowledge, this is the first molecular study on assemblages in *Giardia lamblia* adopted in the Zakho district, province of Duhok, Iraq.

The present study aimed to determine the giardiasis infection rate and identify the assemblages of *Giardia lamblia* in children from four areas in the Zakho district. We collected fecal samples and conducted a microscopic examination.

Genomic DNA was extracted, and assemblage identification was done via amplification of the *gdh* gene using a semi-nested polymerase chain reaction and restriction fragment length polymorphism (RFLP).

Out of 31 *Giardia*-positive samples, 23 were successfully amplified through semi-nested PCR. Nineteen isolates (82.60%) were assigned to assemblage B, and four (17.40%) to assemblage A. Assemblage B was identified as belonging to sub-assemblages B11 and B1V, while assemblage A was identified as sub-assemblages A1 and A11.

This study provides insights about *Giardia lamblia* assemblages in the Zakho district, Duhok province, Iraq, and may serve as a beginning step toward understanding the molecular characterization of *Giardia* in the studied area.

## Introduction

*Giardia lamblia*, a flagellate protozoan parasite, causes human giardiasis. It is more common in places with poor sanitation, but it affects people globally, including in rich nations [1]. Globally, *Giardia lamblia* infections result in around 280 million new cases of human giardiasis per year [2]. In developing countries, the infection rate is between 10% and 50%, especially among younger people in impoverished regions [3]. This microorganism can be found in two different forms: the motile trophozoite stage, which inhabits the host digestive tract, and the latent and environmentally resistant cyst stage, which is responsible for transmission and is dispersed in the environment through the host feces [4]. Cysts have an ovoid form and a diameter of 126 µm. It possesses flagella remnants inside the granular cytoplasm, and the organism has two pairs of nuclei, one clustered at one end and the other in pairs at the other end [5]. Trophozoites have two similar nuclei, a ventral adhesive disk that attaches to intestinal epithelial cells, and two dark-staining median bodies with unclear functions [4].

Giardiasis symptoms can range from acute to chronic diarrhea, and after infection, persistent diseases have been reported. Asymptomatic hosts can still discharge infectious cysts and serve as a disease transmission vector [6]. The clinical signs of *Giardia lamblia* infection range from the lack of pathological signs to the occurrence of these symptoms. The most common symptoms include vomiting, nausea, flatulence, diarrhea, cramping in the stomach, and weight loss. Giardiasis was identified using a variety of techniques, most notably direct microscopic examination of wet swabs and Lugol's iodine solution. However, microscopic examination is not always effective in identifying *Giardia lamblia* because the cysts are occasionally excreted or may be confused with other organisms [7]. Eight Giardia assemblages (A to H) have been discovered according to host specificity [8]. Among the eight assemblages, both humans and animals have two assemblages (A and B), which include dogs, cats, livestock, and wild animals; the next six assemblages (C to H) are virtually entirely distributed among nonhuman hosts, such as beavers, cats, dogs, and cattle [9]. In Duhok province, most of the studies performed on giardiasis restricted themselves to the prevalence and associated risk factors of this parasite in humans, including different ages, genders, places of residence, and the number of family members. So far, there has been no study dealing with the genotyping of this parasite among humans, particularly infants and children. Therefore, the aims of the present study were to determine the rate of infection with giardiasis and the genotypes of *Giardia lamblia* isolates from children in Zakho district, Duhok province.

## Materials and methods

Sample collection and microscopy

This research is part of a cross-sectional study carried out at the Molecular Biology Laboratory in the Biology Department, College of Science at the University of Zakho between August 2021 and July 2023. A total of 504 fecal samples were collected from children of both genders and different ages (1 month to 15 years) in four different places (Zakho General Hospital, Besive 1, and Chamisku camps for internally displaced people; in addition to students of primary schools located in suburban areas) in the Zakho district. The sample size has been taken according to the Cohen method [10]. Each fresh fecal sample was collected in a sterile container after obtaining verbal consent from each child or the accompanied parents for children for the use of the specimen in addition to consent from the general directorate of health in Zakho (700/3, August 11, 2021). Stool samples were assessed microscopically for the existence of parasite cysts or trophozoites through the zinc sulfate flotation techniques developed by Brooke and Melvin [11].

Extraction of DNA

A total of 31 Giardia lamblia-positive samples that had been preserved at −20 °C were subjected to the genomic DNA extraction procedure using the PrestoTM Stool gDNA Extraction Kit (Geneaid, New Taipei City, Taiwan) and following the manufacturer's guidelines. The purity and concentration of the isolated DNA were measured using the Nanodrop Spectrophotometer (Thermo Scientific, Waltham, MA) at absorbance wavelengths of 260 and 280 nm.

Amplification of DNA

The obtained genomic DNA was amplified via semi-nested PCR, utilizing primers targeting gdh genes. The primers utilized were external forward primer (GDHeF: 5ʹ-TCA ACG TYA AYC GYG GYT TCC GT-3ʹ) and internal forward primer (GDHiR: 5ʹ-CAG TAC AAC TCY GCT CTC GG-3ʹ) in the primary round of the PCR reaction. For the second round of PCR, the primers GDHiF (5ʹ-CAG TAC AAC TCY GCT CTC GG-3ʹ) and GDHiR (5ʹ-GTT RTC CTT GCA CAT CTC C-3ʹ) were used as previously described by Read et al. [12]. The amplification reaction was performed in a 25-μl reaction tube consisting of 12.5 µl of Crystal Hot Start Master (2×), 2 µl of (10 pmol/µl) primer (1 µl of forward and 1 µl of reverse), 1 µl of DNA template, and 9.5 µl of deionized distilled water. The amplification was performed using a thermocycler (Gene AMP PCR System 9700 Thermocycler). The PCR cycling protocol was as follows: one cycle of initial denaturation at 94 ˚C for two minutes, annealing at 56 ˚C for one minute, and extension at 72 ˚C for two minutes; then 55 cycles of denaturation at 94 ˚C for 30 seconds, annealing at 56 ˚C for 20 seconds, and extension at 72 ˚C for 45 seconds, followed by one cycle of final extension at 72 ˚C for seven minutes. The amplicons were visualized on a 1.5% agarose gel electrophoresis stained with RedSafe dye. All samples were successfully amplified, yielding fragments of 432 bp.

Restriction fragment length polymorphism (RFLP)

Restriction endonuclease enzymes Nla 1V and Rsa1 were used separately for the digestion of PCR products for the *gdh* gene. These two restriction enzymes were specialized for digestion of certain sequences that were unique to one genotype without the others; the restriction endonuclease enzyme (Nla 1V) was utilized for discriminating genotypes belonging to genotypes (A) and (B), while the restriction endonuclease enzyme (Rsa1) was utilized for distinguishing sub-genotypes [12].

Statistical analysis

We analyzed the data using the Chi-square test using a statistical package for social science (SPSS, version 25, Armonk, NY and GraphPad Prism, version 9.4.1, GraphPad Software, Inc., La Jolla, CA). P-values < 0.05 were considered significant for all statistical analyses.

## Results

**Figure 1:**
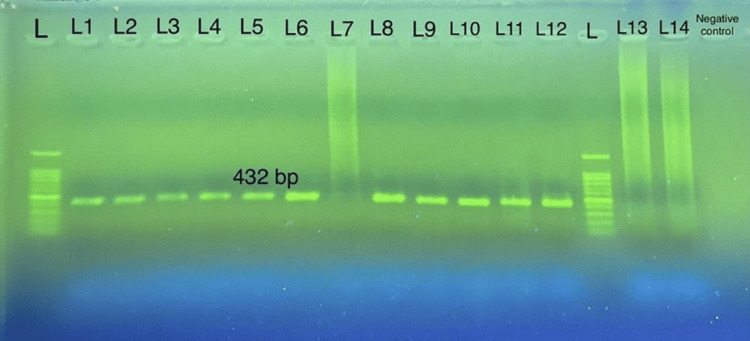
Seminested PCR products of Giardia lamblia from a stool sample obtained with GDeF and GDiR primers on agarose gel (size 432 bp). Lanes L1 to L14: sample number; L: DNA ladder (100–1000 bp).

Microscopic examination revealed that 31 samples out of 504 (6.15%) were identified as positive for giardia (trophozoites or cysts). Additionally, 473 samples out of 504 (93.85%) showed negativity for giardia. Also, the extracted genomic DNA had a minimum concentration of 18.9 ng/µl and a maximum concentration of 304 ng/µl. The purity of the extracted DNA ranged from 1.74 to 1.98 nm, measured at A260 and A280 nm. A fragment of the gdh gene was amplified using GDHeF, GDHiR, and GDHiF primers, resulting in 432 bp of DNA fragments in the second round of semi-nested PCR (Figure 1).

Microscopy revealed 31 stool samples to be positive for Giardia, of which 23 (74.20%) were positive using the PCR targeting gdh loci, while 8 (25.80%) were negative.

**Figure 2:**
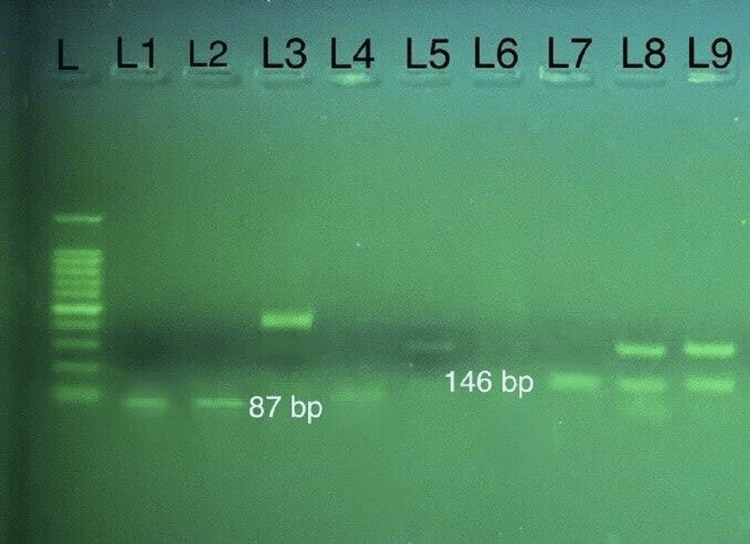
RFLP analysis using the NlaIV restriction enzyme. L represents the DNA ladder (100 bp); lanes 1, 2, and 4 correspond to sub-genotype AII (87 bp), while lane 7 corresponds to sub-genotype AI (146 bp). Lanes 3, 5, 6, 8, and 9 correspond to genotype B (428, 298 bp).

**Figure 3:**
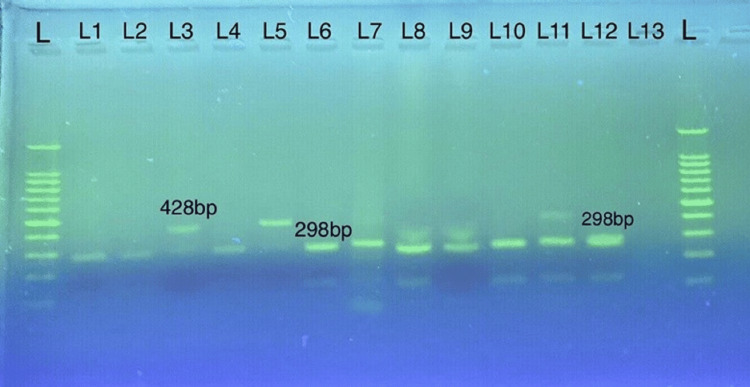
RFLP analysis using the RsaI restriction enzyme. L represents the DNA ladder (100 bp); lanes 3 and 5 correspond to sub-genotype BIV (428 bp), while lanes 1, 2, 4, 6, 7, 8, 9, 10, 11, and 12 correspond to sub-genotype BIII (298 bp). Lane 13 corresponds to the negative control.

The expected 432 bp *gdh* gene fragment was amplified in 23 samples. The assemblage A showed RFLP patterns of 87 and 146 bp, typical for sub-assemblages AII and A1, as seen in Figure 2. On the other hand, assemblage B displayed RFLP patterns of 298 and 428 bp as sub-assemblages BIV and BIII following digestion with NIa1V and RsaI restriction enzymes (Figure 3).

The RFLP analysis exhibited differences between the assemblages. Notably, the highest prevalence was observed in assemblage B, with a rate of 19 (82.60%). Conversely, the lowest prevalence was observed in assemblage A, with a rate of 4 (17.40%), as indicated in Table 1. Statistically, a significant difference was observed in the percentage rates between both assemblages at the probability level (P<0.05).

**Table 1:** Percentage of genotypes of Giardia lamblia isolated from children. *Statistically significant at P<0.05.

Assemblage	Number	Percentage	P value
A	4/23	17.40%	P<0.05
B	19/23	82.60%	

**Figure 4:**
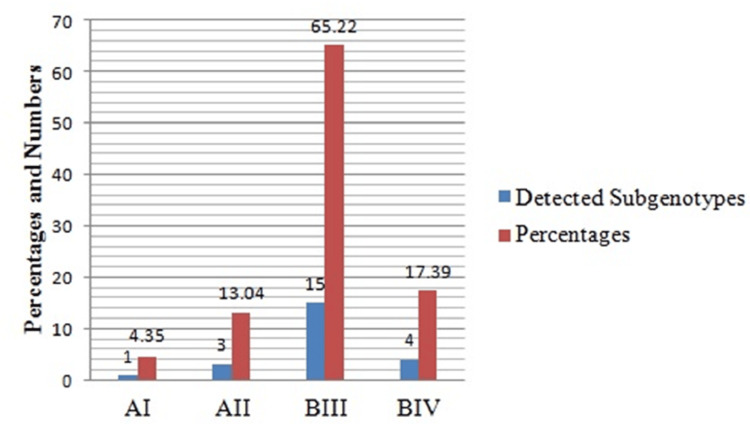
Percentage of sub-genotypes of G. lamblia determined by RFLP analysis.

Figure 4 illustrates the prevalence of identified sub-assemblages. Differences were observed in the rates of sub-assemblages B111 (15, 65.22%) and B1V (4, 17.40%), as well as in sub-assemblages A1 and A11 (1, 4.35%) and (3, 13.04%), respectively. The highest percentage (65.33%) was recorded in sub-assemblage B1111, while the lowest percentage (4.35%) was observed in sub-assemblage A1.

## Discussion

According to 2011 World Health Organization (WHO) data, diarrheal diseases, such as giardiasis, are more prevalent in poor nations and account for a significant portion of childhood morbidity and mortality [13]. In the current investigation, the rate of infection with *Giardia lamblia* in children in Zakho district was 6.15%. This finding is more or less similar to studies done by Khudhair [14] in Erbil province and Chamchamal (Sulaimani province, Iraq) and Al-Marzoqi [15] in Babel province, Iraq, where the percentages of giardiasis were 4.2% and 5.4%, respectively. The current finding defies earlier research on internally displaced people in Kirkuk province conducted by Salman et al. [16], Hussein [17] in Thi-Qar province, Iraq, and Al-Saeed and Issa [18] in Duhok province, Iraq, which found significantly higher rates of Giardia lamblia infection (10.31%, 23.7%, and 38.5%, respectively). These discrepancies could be explained by a variety of factors, including ecological, dietary, geographic, and health-associated factors, the number of samples in the screened investigation, and the diagnostic technique used [19].

Furthermore, the *gdh* gene's amplification percentage from stool samples 23 (74.2%) in this investigation corresponds with and contradicts research done by a number of authors. A stiff wall that prevents the release of DNA from the cysts, low DNA concentrations, PCR inhibitors found in some fecal samples (such as lipids, bile salts, polysaccharides from mucus, bacteria, and food decomposition products), or the degradation of parasites during storage could be the cause of some fecal samples that tested positive under a microscope but produced false negative results when tested by PCR assay [20].

Restriction fragment length polymorphism is a sensitive and efficient analytical technique that can be qualified for providing the level of genotyping discrimination between and within genotypes by amplifying some genes, such as *gdh*, facilitating the detection of mixed genotype presence. It is noteworthy that all genes can be effectively categorized at the genotype level of *Giardia lamblia* isolates; the glutamate dehydrogenase gene facilitates differentiation between genotypes A and B sub-genotypes [21]. Among the most prevalent and practical genetic markers for Giardia lamblia genotyping is the *gdh* gene. It has been verified that molecular analysis of this gene may divide isolates of genotypes A and B into four groups: AI, AII, BIII, and BIV, through the use of the PCR-RFLP approach [22].

Giardia lamblia has a clonal population structure for the detection of sub-genotypes within A and B genotypes by using one set of primers for amplification of the *gdh* locus, inserting two types of restriction enzymes, and obtaining different fragments of molecular length. The intragenotyping variation of *gdh* in assemblages A and B is helpful for sub-genotyping [23]. Sub-genotype BIII is more prevalent among the study groups than other sub-genotypes, with a percentage of 15 (65.22%), according to the study's results. These findings coincide with the research conducted in Al-Diwaniyah City, Iraq, by AL-Mayali and AL-Ibrahimi [24] and Tappeh et al. [25] in the west Azerbaijan province, Iran. However, this result is in contrast with earlier studies conducted in Egypt by Chanu et al. [26] and India by Elhadad et al. [27]. It has been proposed that assemblage A is connected to zoonotic transmission since it is commonly found in a variety of animals, including a large number of cattle [28-30].

Assemblage B was more prevalent among the children in this study, which may suggest that human-to-human transmission is the most likely mode of transmission. In a study involving children in Brazil [31], assemblage B was associated with a higher rate of cyst discharge than assemblage A. This finding could potentially lead to a higher occurrence of assemblage B due to its larger distribution.

Several investigations have found a connection between genotype B symptomatic infection and genotype A asymptomatic infection. In one such investigation done in Spain by Sahagun et al. [32], giardiasis symptoms and genotypes were found to be correlated, with the highest rate of assemblage B infection found in patients who do not exhibit any symptoms and the highest rate of assemblage A infection found in patients who exhibit symptoms but do not show any significant differences in their illness.

There are several limitations regarding the DNA extraction in this investigation. Some stool samples could not be assembled, most likely as a result of the samples' repeated freezing and thawing since their original collection, which could have accelerated the degradation of DNA. The small number of positive samples analyzed (just four assemblages A were found) makes it impossible to compare the characteristics of assemblages A and B in terms of symptomology and sociodemographic factors.

Despite the limitations mentioned above, I think that the detection of Giardia lamblia assemblages and sub-assemblages in children in the Zakho district using one of the most sensitive and reliable DNA markers provided a significant contribution.

## Conclusions

This study illustrated that assemblages A and B in *G. lamblia* are prevalent in children in the Zakho district, with assemblage B predominating, which may suggest that human-to-human transmission is the most likely mode of transmission. It also provides some preliminary data on the assemblage and sub-assemblage distribution of *G. lamblia* among children in Zakho district, Duhok province, Iraq. This is consistent with the body of research that has been done on the mode of transmission in *G. lamblia*. On the other hand, the current study highlighted that restriction fragment length polymorphism analysis of the *gdh* gene is a powerful and efficient marker for the detection of genotypes and sub-genotypes in *G. lamblia*.
